# Minimal access direct spondylolysis repair using a pedicle screw-rod system: a case series

**DOI:** 10.1186/1752-1947-6-396

**Published:** 2012-11-23

**Authors:** Mohamed Mohi Eldin

**Affiliations:** 1 Department of Neurosurgery, Faculty of Medicine, Cairo University, Cairo, Egypt

## Abstract

**Introduction:**

Symptomatic spondylolysis is always challenging to treat because the pars defect causing the instability needs to be stabilized while segmental fusion needs to be avoided. Direct repair of the pars defect is ideal in cases of spondylolysis in which posterior decompression is not necessary. We report clinical results using segmental pedicle-screw-rod fixation with bone grafting in patients with symptomatic spondylolysis, a modification of a technique first reported by Tokuhashi and Matsuzaki in 1996. We also describe the surgical technique, assess the fusion and analyze the outcomes of patients.

**Case presentation:**

At Cairo University Hospital, eight out of twelve Egyptian patients’ acute pars fractures healed after conservative management. Of those, two young male patients underwent an operative procedure for chronic low back pain secondary to pars defect. Case one was a 25-year-old Egyptian man who presented with a one-year history of axial low back pain, not radiating to the lower limbs, after falling from height. Case two was a 29-year-old Egyptian man who presented with a one-year history of axial low back pain and a one-year history of mild claudication and infrequent radiation to the leg, never below the knee. Utilizing a standardized mini-access fluoroscopically-guided surgical protocol, fixation was established with two titanium pedicle screws place into both pedicles, at the same level as the pars defect, without violating the facet joint. The cleaned pars defect was grafted; a curved titanium rod was then passed under the base of the spinous process of the affected vertebra, bridging the loose fragment, and attached to the pedicle screw heads, to uplift the spinal process, followed by compression of the defect. The patients were discharged three days after the procedure, with successful fusion at one-year follow-up. No rod breakage or implant-related complications were reported.

**Conclusions:**

Where there is no evidence of frank spondylolisthesis or displacement and pain does not radiate below the knee, we recommend direct repair of the pars interarticularis fracture, especially in young active adults. We describe a modified form of the Buck screw procedure with a minimally invasive, image-guided method of pars interarticularis fixation. The use of image guidance simplifies the otherwise difficult visualization required for pars interarticularis screw placement and allows minimal skin and muscle dissection, which may translate into a more rapid postoperative recovery.

## Introduction

Lumbar spondylolysis (fatigue fracture of the pars interarticularis) is a radiographic finding, which is common on spine radiographs. Lumbar spondylolysis frequently occurs during childhood. The cause of spondylolysis is repetitive stress on the pars interarticularis (isthmus) of the lumbar vertebrae in the form of loading and unloading on the pars from repetitive spinal motion, especially lumbar flexion extension; and, to a lesser degree, rotation as part of abnormal counter-movements in the low lumbar spine during physical activity. During the acute phase, the majority of symptomatic spondylolysis can be successfully treated conservatively, but those who remain symptomatic may benefit from surgery. *In situ* spinal fusion of the involved level is widely accepted as the treatment of choice for symptomatic. Two-level posterolateral spinal fusion and decompression of the segment is the usual choice of treatment in spondylolysis with chronic back pain. However, the disadvantages of that procedure are loss of motion at the fused segment and an increase in the rate of degeneration of the adjacent unfused segment, especially in the young and active. Direct repair and reconstruction of the pars defect, in cases without degenerative changes, is a logical and less aggressive approach, and has the advantage of maintaining the motion segment, with compression across the bone-grafted defect to enhance and ensure better fusion [1-3].

Symptomatic spondylolysis is always challenging to treat because the pars defect causing the instability needs to be stabilized but segmental fusion needs to be avoided. Direct repair of the pars defect is ideal in cases of spondylolysis in which posterior decompression is not necessary. The goal of the pars repair is to obtain fusion of the defect, to restore the anatomy and stability of the spine, to preserve the mobility of the segment, and to prevent later development of slippage or adjacent segment failure. Kimura, in 1968, was the first to develop this concept as an alternative to segmental fusion. Since then, several non-fusion alternative techniques have been described in an attempt to directly repair the pars defect. Available techniques include either direct osteosynthesis across the pars defect with a lag screw or indirectly applying compression across the defect using a combination of wires, hooks, pedicle screws and rods. Although the various techniques have not been compared with regards to clinical or radiological outcome, the overall clinical outcome seems to be encouraging, especially in terms of quality of life. Those techniques were mainly used in young populations: children, adolescents and young adults. Good results using these techniques with young people without spondylolisthesis, facet arthritis or degenerative disc disease have been reported. Deguchi *et al*. compared the biomechanics performance of these various fixation techniques and found that the pedicle-screw-hook system brings a greater biomechanical stability to the defect during motion, hence better fusion [1].

The present report shows the clinical results using segmental titanium pedicle-screw-rod fixation with bone grafting in patients with symptomatic spondylolysis, a modification of a technique first reported by Tokuhashi and Matsuzaki in 1996 [2]. We also describe the surgical technique, assess the fusion and analyze the outcomes of patients. According to Louis’ criteria, we carried out the surgery in patients with moderate disc signal modification, as demonstrated on T2-weighted magnetic resonance imaging (MRI) [3]. Pfirrmann’s classification was used to assess the vertebral disc signal and the limit of reconstruction was set at grade 3 [4].

## Case presentation

Over two years at Cairo University Hospital, eight out of 12 Egyptian patients had acute pars fractures that healed after conservative management. Four of these patients had continued chronic stress defects that were also managed conservatively, with no improvement in two cases who had been surgically treated. None of our patients showed other radiological anomalies, such as spina bifida occulta, or a lumbosacral anomaly.

We evaluated two young male patients who underwent the operative procedure for chronic low back pain secondary to pars interarticularis defect. Both patients had low back pain; one also had infrequent radiation to the leg, never below the knee. On clinical examination, pain was experienced, in both patients, on extension and lateral deviation, and full flexion was possible with no discomfort. Diagnostic infiltration of the pars defect with lidocaine 2% was used in these two cases to detect the pain source and to make sure that no other causes might have been missed.

Preoperative imaging included lumbosacral MRI to assess the diseased segments and to evaluate potential contraindications for the approach, for example, degenerated disc, frank instability,or multilevel disease. Flexion-extension standing lateral X-ray scans were used to evaluate segmental mobility. Our patients were selected on the basis of imaging evidence of spondylolysis with or without minimal-grade spondylolisthesis and axial pain. Neither patient had abnormal neurological signs, or evidence of nerve-root compression. The lesion was located at the fifth lumbar vertebra bilaterally in one patient with minimal-grade spondylolisthesis, and at the fourth lumbar vertebra bilaterally in the other, with no slippage.

**Figure 1:**
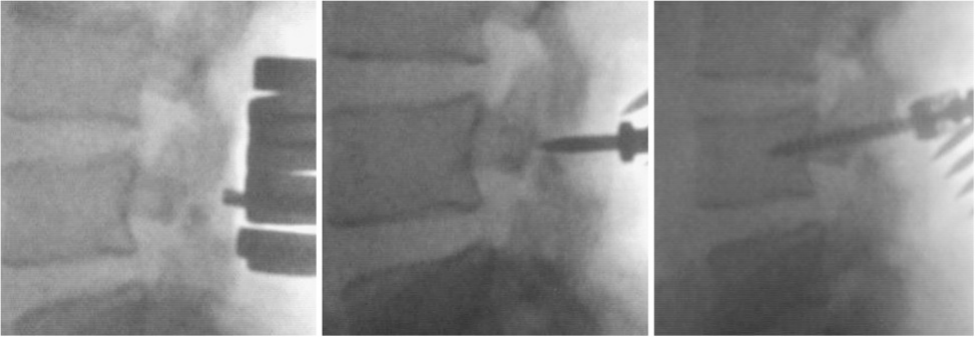
Intraoperative fluoroscopic images of the steps for a percutaneous technique of radiological guidance for screw insertion, without violating the facet joint.

**Figure 2:**
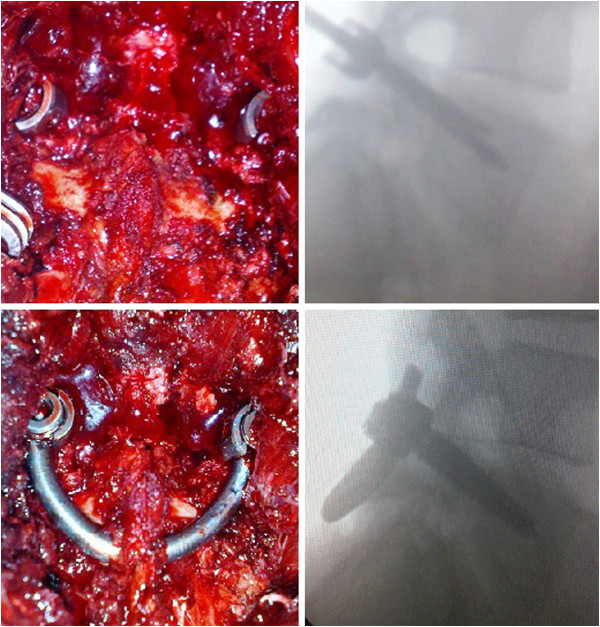
Intraoperative view of the procedure (left) and lateral fluoroscopic images depicting screws insertion into the L4 pedicles (right) and intraoperative view of the construct.

**Figure 3:**
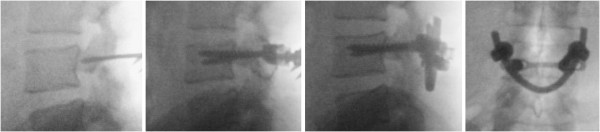
Intraoperative fluoroscopic images of the steps of the procedure (left to right), showing the defect (left) and screw insertion into the L4 pedicles with intraoperative posteroanterior view of the construct (right).

The operations (Figures 1, 2 and 3) were performed at Cairo University. A standardized mini-access fluoroscopically guided surgical protocol was used for each case. Our patients were positioned on a flexion frame in the prone position, with both hips extended to maintain the lumbar lordosis. The operative table was translucent, with space for the C-arms underneath at the level of the lumbosacral spine. A minimal access midline incision was made, centered over the disc space of interest. Posterior subperiosteal dissection was performed exposing the involved vertebra, directly cranial to the fracture site, under fluoroscopic guidance. Care was taken not to violate the facet capsule. The defect in the pars was localized bilaterally. Fixation was established with two top-loading titanium pedicle screws placed into each pedicle, at the same level as the pars defect, using a percutaneous technique of radiological guidance of screw insertion, without violating the facet joint.

The pars defect cleaning began with debridement curettage of all soft and cartilaginous tissue and all callus sclerotic bone from the defect until bleeding bone was evident. The pars defect was then grafted with a cancellous bone autograft that was packed into the defect. A curved titanium rod was bent and passed under the base of the spinous process of the affected vertebra, bridging the loose fragment. It was then attached to the titanium pedicle screw heads on both sides at the involved level to uplift the spinal process, followed by compression of the defect. Once the rod was advanced to its final position it was tightened and crimped. Both pedicle screws were tightened to compress the pars defect bone grafts. The pedicle screw caps were tightened and the small incision closed in layers.

**Figure 4:**
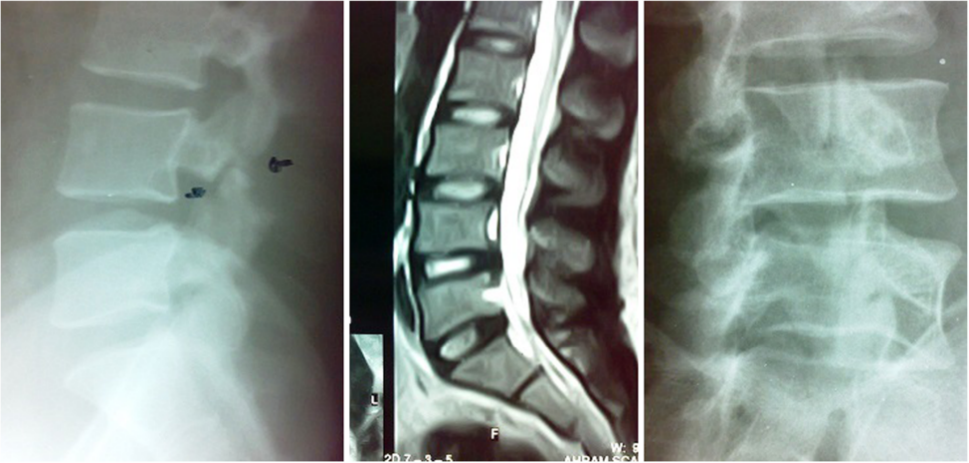
Computed tomography assessment of the union at three and six months (left, center) and postoperative lateral image of the construct (right) showing apparent bony union of the defect.

Postoperatively, our patients were placed flat on their backs for three to five days after the operation. Their activity was limited for four to six weeks, and a lumbosacral corset worn. When a lumbar brace was applied, full mobilization was allowed. Sedentary work, driving, swimming, isometric exercises and bicycle riding were allowed four weeks after the operation. The brace was removed after three months. The first computed tomography (CT) assessment of the union was at three months (Figure 4). Where union was not apparent at three months a further scan was performed six months after surgery, by which time union had occurred. Once oblique CT scanning showed evidence of union, the patients were allowed to resume full activity. Strenuous work and activity were not recommended until after six months. Both patients showed successful fusion at a one-year follow-up, and had marked improvement in preoperative pain. No rod breakage or implant-related complications were reported during the follow-up period.

### Case one

**Figure 5:**
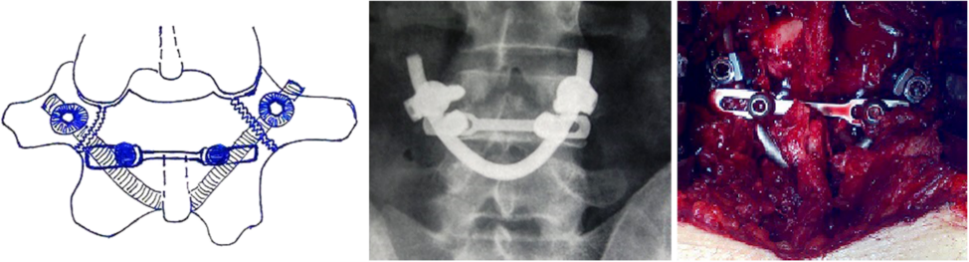
Lumbar midline sagittal T2-weighted magnetic resonance imaging (center) of Case one showing no degenerated disc, frank instability, or multilevel disease; and extension standing lateral and oblique X-rays (left, right) showing the pars defect with no segmental instability.

**Figure 6:**
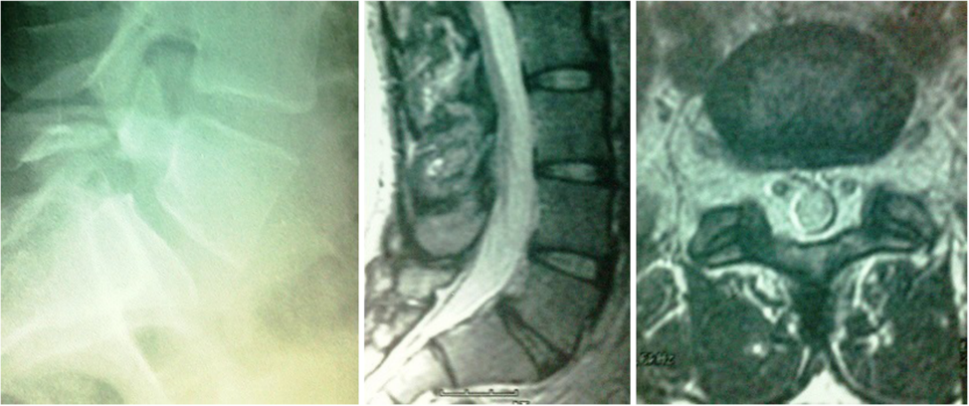
A graphic representation of the procedure (left) in Case one and anteroposterior fluoroscopic images depict screw insertion into the L4 pedicles (center), and intraoperative view of the construct (right).

**Figure 7:**
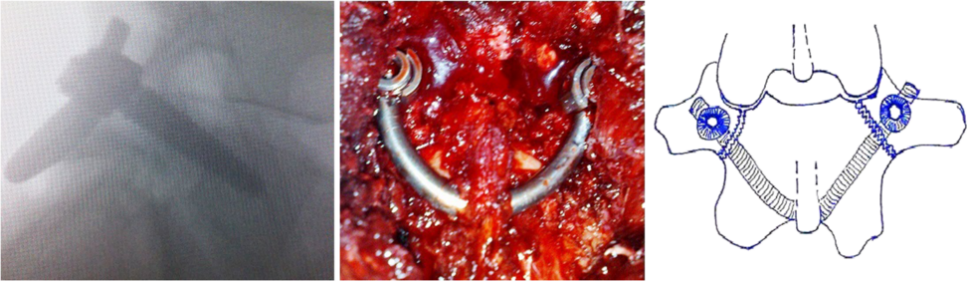
Intraoperative fluoroscopic lateral image (left) and operative view of the construct (center) and a graphic representation of the procedure (right).

Case one was a 25-year-old Egyptian man, known to be a cigarette smoker, who presented with a one-year history of axial low back pain, not radiating to his lower limbs, after falling from height. His pain was mechanical, exacerbated by standing or walking for extended periods of time and improved by lying down, and refractory to extensive conservative treatment. During a physical examination our patient showed no sensory-motor deficits. A lumbar MRI showed evidence of L4 spondylolysis with no spondylolisthesis or foraminal stenosis (Figure 5). The operative time was 120 minutes, and blood loss was minimal (50mL). Our patient was ambulatory after surgery and reported relief from back pain (maximal pain severity of 10 at pretreatment reduced to three after treatment), improvements in back function (from 68% to 15% on the Oswestry Disability Index), and no complications. Our patient was discharged from the hospital two days after the procedure. To confirm the adequate placement of the instrumentation, as shown in the graphic representation (Figure 6), and to accurately evaluate the final constructs, a CT scan was obtained after the operation. Successful fusion, defined as no motion at the treated segment on flexion or extension radiographs and evidence of bone growth between the adjacent vertebral bodies on reconstructed CT images, was demonstrated at up to one-year follow-up (Figure 7).

### Case two

**Figure 8:**
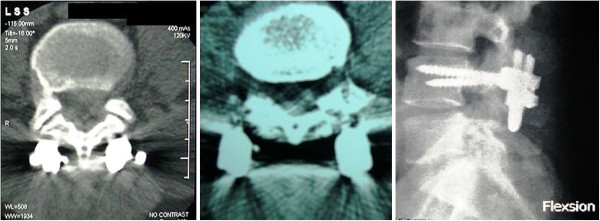
Lumbar midline sagittal and axial T2-weighted magnetic resonance imaging (center, right) showing no degenerated disc, neural compromise, frank instability, or multilevel disease; and extension standing lateral and oblique X-rays (left) showing the pars defect with minimal forward slippage.

Case two was a 29-year-old Egyptian man who presented with a one-year history of axial low back pain and a one-year history of mild claudication and infrequent radiation to his leg, never below his knee. The pain was described as nine out of 10 on average and 10 out of 10 at its worst, of mechanical type and refractory to conservative treatment. During a physical examination our patient showed no sensory-motor deficits. He had tender sacroiliac joints bilaterally. A lumbar MRI showed evidence of L5 spondylolysis with minimal-grade L5 to S1 spondylolisthesis with no foraminal stenosis (Figure 8). The operative time was 130 minutes, and blood loss was minimal (50mL). Our patient was ambulatory after surgery and reported relief from back pain (from 10 to two), resolution of pseudo-radicular symptoms (from 10 to one), improvements in back function (from 65% to 10% on the Oswestry Disability Index), and no complications. Our patient was discharged from the hospital three days after the procedure with successful fusion at one-year follow-up.

## Discussion

During the acute phase, the majority of symptomatic spondylolysis can be successfully treated conservatively, but those who remain symptomatic may benefit from surgery. Early diagnosis is an important factor for a good prognosis in bone healing. Morita *et al*. studied 185 adolescents with spondylolysis and classified the pars defects into early, progressive and terminal stages [1,5]. Conservative management produced healing in 73% of the early stage cases, in 38.5% of the progressive stage cases and in 0% of the cases with terminal defects, which more probably required surgical intervention. Wiltse *et al*. demonstrated that 12 of 17 young patients diagnosed with spondylolysis showed osseous healing with conservative treatment and no surgery [6]. Blanda *et al*. examined 62 patients with spondylolysis and found that 52 patients had excellent results with conservative treatment, with an average follow-up of 4.2 years [7]. These studies suggest that spondylolysis can be successfully treated using conservative treatment if diagnosed at an early stage. It must be emphasized that in most of the patients significant symptoms never develop and that the defect *per se* is not an indication for surgical intervention. In the symptomatic group, the vast majority of patients can be treated conservatively.

In the small group of patients not responding to conservative management, we believe there is an indication for surgery. Ideally, direct pars repair is recommended for young patients with spondylolysis who have back pain alone, have no signs of neurological involvement, do not respond to conservative treatment over one or more years, have relatively normal discs as seen on MRI, and have pain provocation with a pars block. For patients with a minimum of forward slipping and with an intact disc, restoration by direct repair of the lesion in the pars interarticularis is a more logical step. Like Buck, and Buring and Fredensborg, we have accepted a forward slip of not more than 2mm to 3mm as seen on lateral standing roentgenograms [8,9]. Only patients who had significant pain relief from wearing a plaster-corset were considered candidates for surgery, and only those who had spondylolysis with or without minimal spondylolisthesis were accepted.

Pars repair has been described in many studies using many techniques: Kimura in 1968, Buck’s screw fixation in 1970, Morscher *et al.* with hooks and screws in 1984, Scott’s transverse process wiring in 1986, and others [10-12]. The difficulty is to create a sufficient compression and strong fixation to the pars defect to allow the bony ingrowth of the bone graft without breaking the lamina or the transverse process, and without a nerve injury or irritating the facets. We agree with Hardcastle *et al*. [13] that fusion of the defect in the pars interarticularis attempts to restore normal anatomy and to retain movement at the involved level. Two techniques have been described: tension-band wiring [14-16] and screw fixation [8].

In this case report of a technical note, we are presenting a pedicle screw-based technique with a bended rod and autologous bone graft. The intraoperative picture and postoperative X-ray show the precise type of surgery, with excellent over-bridging of the defect zone, confirmed by CT. The clinical outcome seems to be good, with improvement in the pain level and functional status of the patients and progress during follow-up.

The technique with two pedicle screws and bended rod was also published by Ulibarri *et al*. [17] in a cadaver analysis and clinical study on five patients with a follow-up of 4.6 years. The biomechanical findings were promising and the clinical results were good with modified Oswestry scores of 0% to 13%.

The presented technical note is one of a series of developments of pars repair techniques. For spondylolysis without or with minimal spondylolisthesis at any level, we use a pedicle screw-rod construct with compression and bone grafting at and around the defect. This technique offers the following advantages over Buck’s procedure: more stiffness to flexion loads, larger area available for bone grafting, and less need for postoperative immobilization. Our modification, with a two-piece construct of rod and cross-bar, seems to be a smart solution to this challenging problem, especially in cases with minimal displacement, in the L5 to S1 level, and in active adults. The cross-bar increases the number of fixation points and thus increases the overall strength of the construct, in addition to ensuring compression of the defect to enhance fusion. Therefore, this technique is useful for fusion of the pars defect and restoration of normal motion of the lumbosacral spine in patients with spondylolysis and a minimal degree of isthmic spondylolisthesis.

Our results show that surgery enables a fast return to full activity. Neither of our patients had pain radiating below the knee; one had very minor spondylolisthesis. Both were under 32 years of age, confirming that good results are achievable in this age group [16]. Long-term results are good; both Buck [8] and Roca *et al*. [15] reported no late breakdowns in non-sporting patients. Our patient with the longest follow-up (three years) continues without back pain and with no late breakdowns.

The definition of treatment success typically includes the resolution of symptoms, return to work or fusion across the defect as the primary outcome. In discussing the successful outcomes, we advise direct surgical intervention in patients younger than 30 years of age. Despite excellent operative outcomes and a return to activity in our patients, this approach needs to gain popularity. A minimal access approach is used to place this type of construct, with minimal tissue disruption, and this technique compresses the defect directly and ultimately helps new fusion to take place. Using image guidance, we were able to fix the pars interarticularis with minimal tissue damage, allowing the fracture to properly heal in a healthy mobile segment.

## Conclusion

Spondylolysis is often asymptomatic, but occasionally may produce back pain that does not respond to conservative treatment and continues to a more chronic illness. When conservative treatment fails to produce improvement, surgical repair of the defect may allow a return to full normal daily activity. Where there is no evidence of frank spondylolisthesis or displacement and pain does not radiate below the knee we recommend direct repair of the pars interarticularis fracture, especially in young active adults.

We described a modified form of the Buck screw procedure with a minimally invasive, image-guided method of pars interarticularis fixation. The use of image guidance simplifies the otherwise difficult visualization required for pars interarticularis screw placement and allows minimal skin and muscle dissection, which may translate into a more rapid postoperative recovery. More applications of our technique may allow such uncommon hardware configuration to be both successful and less invasive.

## Consent

Written informed consent was obtained from the patients for publication of this case series and any accompanying images. A copy of the written consent is available for review by the Editor-in-Chief of this journal.

## Competing interests

The author declares that he has no competing interests.
